# Gut Microbiota as a Target for Preventive and Therapeutic Intervention against Food Allergy

**DOI:** 10.3390/nu9070672

**Published:** 2017-06-28

**Authors:** Rosita Aitoro, Lorella Paparo, Antonio Amoroso, Margherita Di Costanzo, Linda Cosenza, Viviana Granata, Carmen Di Scala, Rita Nocerino, Giovanna Trinchese, Mariangela Montella, Danilo Ercolini, Roberto Berni Canani

**Affiliations:** 1 Department of Translational Medical Science-Pediatric Section, University of Naples “Federico II”, 80131 Naples, Italy; aitoro.rosita@gmail.com (R.A.); paparolorella@gmail.com (L.P.); antonioamoroso87@gmail.com (A.A.); mara.dicostanzo@live.it (M.D.C.); lindacosenza@libero.it (L.C.); vivianagranata@gmail.com (V.G.); carmendiscala@gmail.com (C.D.S.); ritanocerino@alice.it (R.N.); giovanna.trinchese@unina.it (G.T.); mariangelamontella@libero.it (M.M.); 2 Department of Agricultural Sciences, Division of Microbiology, University of Naples “Federico II”, 80055 Portici, Italy; ercolini@unina.it; 3 Task Force on Microbiome Studies, University of Naples “Federico II”, 80131 Naples, Italy; 4 European Laboratory for the Investigation of Food Induced Diseases, University of Naples “Federico II”, 80131 Naples, Italy; 5 CEINGE Advanced Biotechnologies, University of Naples “Federico II”, 80131 Naples, Italy

**Keywords:** cow’s milk allergy, diet, immune tolerance, dysbiosis, probiotics, short chain fatty acids, butyrate

## Abstract

The gut microbiota plays a pivotal role in immune system development and function. Modification in the gut microbiota composition (dysbiosis) early in life is a critical factor affecting the development of food allergy. Many environmental factors including caesarean delivery, lack of breast milk, drugs, antiseptic agents, and a low-fiber/high-fat diet can induce gut microbiota dysbiosis, and have been associated with the occurrence of food allergy. New technologies and experimental tools have provided information regarding the importance of select bacteria on immune tolerance mechanisms. Short-chain fatty acids are crucial metabolic products of gut microbiota responsible for many protective effects against food allergy. These compounds are involved in epigenetic regulation of the immune system. These evidences provide a foundation for developing innovative strategies to prevent and treat food allergy. Here, we present an overview on the potential role of gut microbiota as the target of intervention against food allergy.

## 1. Introduction

During the last several decades, a changing patterns in the epidemiology of food allergy [FA] have been observed, with an increased prevalence, severity of clinical manifestations, and risk of persistence until later ages [1]. Atopic family history, ethnicity, atopic dermatitis (AD), and related genetic polymorphisms have been associated with FA development [2]. Although genetic factors may predispose individuals to the development of FA among selected individuals, they cannot explain the changes in epidemiology over this short time frame, suggesting that environmental factors promote FA [3]. FA develops following loss of immune tolerance, which results in allergic sensitization and subsequent disease manifestation and progression.

The initial exposure to food allergens occurs predominantly via the gastrointestinal tract or skin. An impaired skin barrier could lead to increased transcutaneous passage of antigens and subsequent sensitization. An association between the early onset of AD and development of FA has been shown [4]. In the gastrointestinal tract, the two main factors influencing immune tolerance are dietary factors and microbiota composition and function [5]. Kim et al. demonstrated that under normal physiological conditions, macromolecules from the diet induce the bulk of regulatory T cells (Tregs) development, which is essential for suppressing a default immune response to dietary antigens [5]. Observational studies have suggested that the early introduction of peanut [6], egg [7], or cow’s milk [8] may prevent the development of allergy to these foods. A randomized controlled trial (Learning Early about Peanut Allergy, LEAP) showed that the early consumption of peanut in high-risk infants with severe eczema, egg allergy, or both reduced the development of peanut allergy by 80% by 5 years of age [9]. The Persistence of Oral Tolerance to Peanut (LEAP-On) study showed that the absence of reactivity is maintained in these subjects [10].

The gut microbiota could be defined as the trillions of microbes that collectively inhabit the gut lumen [4,11], and increasing evidence shows that altered patterns of microbial exposure [dysbiosis] early in life can lead to FA development by negatively influencing immune system development [12]. Thus, the gut microbiota could be considered a potential target for preventive and therapeutic intervention against FA. Recent studies have reported the efficacy of intervention in the gut microbiota against FA.Here, we review the current understanding of the potential role of gut microbiota as potential target against FA.

## 2. Importance of Microbial Exposure for the Development of Immune Tolerance

**Figure 1:**
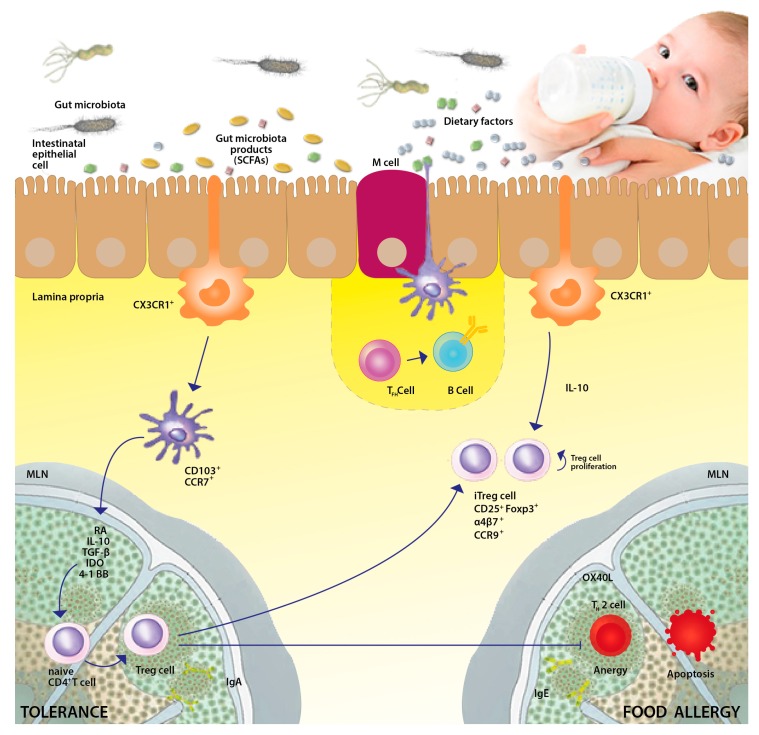
Immune tolerance network in the intestinal lumen: interaction between microbiome and the gut immune system in early-life. The immune tolerance network is mainly composed by the well-modulated activity of different components: gut microbiota (without gut microbiota, it is not possible to achieve oral tolerance); dietary factors (mainly dietary peptides, as amino acids are unable to drive immune tolerance); epithelial cells; dendritic cells; and regulatory T cells. Food antigens and intestinal microbiota constitute the majority of the antigen load in the intestine. CX3CR1^+^ cells (likely macrophages) extend dendrites between intestinal epithelial cells, sample antigens in the gut lumen, and transfer captured antigens via gap junctions to CD103^+^CCR7^+^ dendritic cells (DCs). This subset of DCs migrates from the lamina propria to the draining lymph nodes, where the DCs express transforming growth factor-β (TGFβ), retinoic acid (RA), interleukin-10 (IL-10) and also express the enzyme indoleamine 2,3-dioxygenase (IDO), thereby inducing naïve CD4^+^ T cells to differentiate into regulatory T (Treg) cells. Macrophages also appear to secrete IL-10, leading to Treg cell proliferation. Treg cell express integrin α4β7, which results in homing to the gut where Treg cells may dampen the immune response. CD103^+^ DCs also sample antigens that pass through the epithelial barrier via M cell-mediated transcytosis or by extending a process through a transcellular pore in an M cell. Recent evidence suggests a role for regulatory B cells in activating Tregs after stimulation with microbial factors recognized by Toll-like receptors. B cell clones expressing antibodies specific for food allergen may undergo isotype switching in secondary lymphoid organs with the aid of follicular T helper (TFH) cells. Food tolerance is associated with IgA. For a broader prospective, the complex interaction between intestinal contents and immune and non-immune cells creates an environment that favors tolerance by the inducting IgA antibodies and Tregs, which produce IL-10, a molecule crucial for the induction of tolerance to food antigens.

Immune tolerance is the state of unresponsiveness of the immune system to substances or tissues that have the potential to induce an immune response. Tolerance is achieved through both central tolerance and peripheral tolerance mechanisms [13]. The exact mechanisms involved in the development of immune tolerance have not been not fully defined [14]. Current evidence suggests that the gut microbiota and its metabolites (mainly short chain fatty acids), together with to exposure to dietary factors in early life, critically influence the establishment of immune tolerance to food antigens [5] (Figure 1). Germ-free mice are unable to achieve immune tolerance to food antigens [15]. During the early stage of post-natal life, development of the gut microbiota parallels maturation of the immune system [16]. During vaginal delivery, infants receive their first bacterial inoculum from the maternal vaginal tract, skin tissue, and often fecal matter, exposing the immature immune system of newborns to a significant bacterial load [17]. Maturation of a healthy gut microbiota in early life allows for a change in the Th2/Th1 balance, favoring a Th1 cell response [18], while dysbiosis alters host-microbiota homeostasis, favoring a shift in the Th1/Th2 cytokine balance toward a Th2 response [19]. Gut microbes induce the activation of Tregs which are depleted in germ-free mice [20]. Microbiota-induced Tregs express the nuclear hormone receptor RORγt and differentiate along a pathway that also leads to Th17 cells; while in the absence of RORγt in Tregs, there is an expansion of GATA-3-expressing Tregs, as well as conventional Th2 cells, and Th2-associated pathology is exacerbated [21]. Moreover, it has been demonstrated that under normal physiological conditions, macromolecules obtained via the diet induce Treg cell development in the small intestinal lamina propria, which is essential for suppressing the default strong immune response to dietary antigens [5]. The presence of both diet- and microbe-induced populations of Treg cells may be required to induce complete tolerance to food antigens [5].

It has been speculated that microbiota can activate MyD88 signaling in the lamina propria and follicular dendritic cells (DCs) [22]. Mucosal plasma cells, upon induction by DCs, produce secretory IgA (sIgA). The sIgA system is considered important in the pathogenesis of FA. Delayed development of IgA-producing cells or insufficient sIgA-dependent function at the intestinal surface barrier appears to contribute substantially to FA [21]. This agrees with previous study of minor dysregulations of both innate and adaptive immunity (particularly low levels of IgA) in children with multiple FAs [23]. Furthermore, the gut microbiota stimulates DCs in the Peyer’s patches to secrete transforming growth factor (TGF)-β, C-X-C motif chemokine ligand 13, and B-cell activating protein, which leads to IgA production and class switching [24].

Accordingly, it has been recently demonstrated that dietary elements, including fibers and vitamin A, are essential for the tolerogenic function of CD103^+^ DCs and maintenance of mucosal homeostasis, including IgA production and epithelial barrier function [25].Moreover, in experimental studies, some mice are protected from the development of FA (non-responders) compared with animals showing marked systemic FA symptoms after immunizations [26,27]. This differential immune response is associated with a distinct microbiota composition in mice with a non-responding phenotype [28]. Recent findings have also suggested that neonatal gut microbiome dysbiosis promotes CD4^+^ T cell dysfunction associated with allergy [29] and supports age-sensitive interactions with microbiota [30]. Early-life may be a key “window of opportunity” for intervention given the age-dependent association of the gut microbiome and FA outcomes [31].The microbiota also promotes B cell receptor editing within the lamina propria upon colonization [32]. Regulatory B (Breg) cells are characterized by their immunosuppressive capacity, which is often mediated by interleukin (IL)-10 secretion, but also IL-35 and TGF-β production [33]. An additional immunoregulatory role is the up-regulation of IgG4 antibodies during differentiation to plasma cells. Several studies have demonstrated a potential role for Breg in the induction and maintenance of the tolerance mechanism [34,35,36]. Several types of Bregs with distinct phenotypic characteristics and mechanisms of suppression have been described [34,35,36]; therefore, additional studies are necessary to understand the effective role of Bregs in oral tolerance.In addition, there is a body of data reporting the activation of non-immune pathways in food oral tolerance. Data suggest that a healthy gut microbiota may protect against allergic sensitization by affecting enterocyte function and regulating its barrier-protective properties. Similarly, innate lymphoid cells (ILCs) that are abundant in mucosal and barrier sites are involved in these defence mechanisms [37]. While several subsets of ILCs have been identified, particular attention has been given to ILC3 and its interactions with the microbiota. Among other factors, these cells produce IL-22, a cytokine of central importance in maintaining tissue immunity and physiology via its pleiotropic action in promoting antimicrobial peptide production, enhancing epithelial regeneration, increasing mucus production, and regulating intestinal permeability [38]. How the microbiota affects the turnover of ILC3 remains unclear, but recent evidence supports that defined commensals preferentially impact this subset. Particularly, Clostridia-induced IL-22 has been demonstrated to be an innate mechanism by which the microbiota can regulate the permeability of the epithelial barrier and contribute to protection against food allergen sensitization [15]. In contrast, gut microbiota dysbiosis induces alterations in intestinal epithelial function resulting in aberrant Th2 responses toward allergic, rather than tolerogenic, responses [39].

## 3. Gut Microbiota in FA

**Figure 2:**
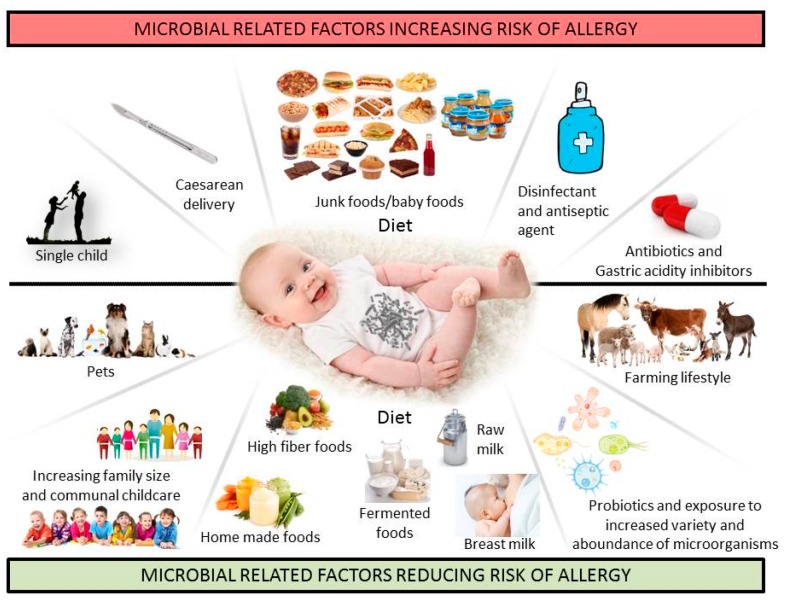
Environmental and lifestyle factors related to microbial exposure and their putative effect on the risk of developing food allergy.

Epidemiological studies have established a correlation between factors that disrupt the microbiota during childhood and immune and metabolic conditions later in life. Several factors responsible for dysbiosis have been associated with the occurrence of FA, such as caesarean delivery [40], lack of breast milk [41], drug use (mainly antibiotics and gastric acidity inhibitors) [42], antiseptic agent use, and low fiber/high fat diet [43] (Figure 2). Emerging data from human studies link the use of antimicrobial agents to the increasing prevalence of FA. Neonatal antibiotic treatment reduced microbial diversity and bacterial load in both fecal and ileal samples and enhanced food allergen sensitization [15]. Even low-dose early-life antibiotic exposure can lead to long-lasting effects on metabolic and immune responsiveness [44]. Maternal use of antibiotics before and during pregnancy, as well as antibiotic courses during the first months of life, are associated with an increased risk of cow’s milk allergy (CMA) in infants [45].

Data characterizing the microbiota of patients with FA are still preliminary because of multiple environmental stimuli that profoundly influence the composition of the gut microbiota [46]. Some studies have failed to identify differences in infant microbiota according to later allergic status, or have found different changes in gut microbiota depending on the cases and groups of subjects. Although compelling evidence for the association of gut microbiota dysbiosis with FA is emerging, heterogeneities in study design, including sampling time points, methods used to characterize the microbiota, and different allergic phenotypes under study, make it difficult to establish a clear correlation between specific bacterial taxa and allergy development. To better identify microbiota changes associated with the emergence of FA, well-phenotyped birth cohorts are needed with long-term follow up.

First studies using bacterial cultures showed that infants allergic to cow’s milk had higher total bacteria and anaerobic counts [47]. There was no association between culturable bacteria and food sensitization by 18 months of age in three cohorts of European infants [48]. Kendler et al. found no association between culturable gut bacteria and sensitization to food including milk, egg, peanut, and hazelnut [49].Pyrosequencing technology can identify approximately 80% more bacteria in the gut than those identified by conventional culture-based methods, revealing the high complexity and diversity of the gut microbiota.Recent evidence suggests that gut dysbiosis precedes FA and influences during early life affected the subsequent development of allergic disease [50]. Nakayama et al. profiled the fecal bacteria compositions of allergic and non-allergic infants and correlated changes in gut microbiota composition with allergy development in later years [51]. They found that in the allergic group, the genus *Bacteroides* at 1 month and genera *Propionibacterium* and *Klebsiella* at 2 months were more abundant, while the genera *Acinetobacter* and *Clostridium* at 1 month were less abundant than in the non-allergic group [51]. Additionally, the relative abundance of total *Proteobacteria*, excluding genus *Klebsiella*, was significantly lower in the allergic than in the non-allergic group at the age of 1 month. Allergic infants with high colonization of *Bacteroides* and/or *Klebsiella* showed less colonization of *Clostridium* within the major phylotypes, suggesting antagonism between these bacterial groups in the gut. *Bacteroides* are sensitive to short-chain fatty acids (SCFAs), particularly under low pH conditions [52], suggesting that the observed antagonism is attributable to an SCFA produced by *Clostridium* [52]. Azad et al. found that an increased *Enterobacteriaceae/Bacteroidaceae* ratio and low *Ruminococcaceae* (*Clostridia* class) abundance, in the context of low gut microbiota richness in early infancy, are associated with subsequent food sensitization, suggesting that early gut dysbiosis contributes to subsequent development of FA [53]. A low level of microbial diversity with reduced *Clostridiales*, and increased *Bacteroidales* have been also observed in the gut microbiota of allergic patients [54].

Cross-sectional studies comparing the intestinal microbial composition of food allergies in healthy subjects have also been performed. Fecal microbial composition was assessed using 16 S rRNA sequencing to determine the differences between children with FA (*n* = 17 with IgE-mediated FA, *n* = 17 with non-IgE-mediated FA) and healthy controls (*n* = 45) [55]. There was no difference in microbial diversity between groups. Subjects with IgE-mediated FA showed increased levels of *Clostridium sensu stricto* and *Anaerobacter* (*Clostridia* class) and decreased levels of *Bacteroides* and *Clostridium* XVIII. Levels of *C. sensu stricto* were also correlated with the levels of IgE [56]. Chen et al. recently showed that children with food sensitization in early life have an altered fecal microbiota and lower microbiota diversity compared to healthy controls. Children with food sensitization showed significantly decreased numbers of *Bacteroidetes* and a significantly increased number of *Firmicutes* compared to healthy children. The most differentially abundant taxa in children with food sensitization were characterized by increased abundances of *Clostridium* IV and *Subdoligranulum* (*Clostridia* class) and decreased abundances of *Bacteroides* and *Veillonella* (*Clostridia* class) [56]. Recently, enriched taxa from the *Clostridia* class and *Firmicutes* phylum were observed in children with a more favourable CMA disease course [57]. Accordingly, a low abundance of some sub-taxa belonging to *Clostridia* may be associated with the development of FA. The *Clostridia* class has become one of the largest genera of bacteria, and presently contains more than 100 species. Some *Clostridia* groups possess pathogenic species; however, most *Clostridia* have a commensal relationship with the host [58].In agreement with this view, a pivotal study by Atarashi et al. showed that the spore-forming component of gut microbiota, particularly clusters IV and XIVa of the genus *Clostridium*, promoted Tregs accumulation in the colonic mucosa. Colonization of mice by a defined mix of *Clostridium* strains provided an environment rich in TGF-β and affected the number and function of colonic Tregs expressing the Foxp3 transcription factor (Foxp3^+^ Tregs) [59]. Foxp3^+^ Tregs play a critical role in oral tolerance [60]. In a subsequent study, Atarashi et al. isolated 17 strains within *Clostridia* clusters XIVa, IV and XVIII from a human fecal sample and demonstrated that these strains affect Tregs differentiation, accumulation and function in mouse colon [61].

Many bacterial metabolites are an important communication tools between the host immune system and commensal microbiota, establishing a broad basis for mutualism [62]. Among these, SCFAs are among the most abundant, and play a critical role in mucosal integrity, and local and systemic metabolic function, and stimulate regulatory immune responses [63,64,65]. *Clostridia* species belonging to cluster IV and XIVa are prominent source of SCFAs in the colon. SCFAs have been implicated in the regulation of both the proportions and functional capabilities of colonic Tregs [62], which, in some studies, has been specifically attributed to butyrate production by spore-forming *Clostridiales* [63]. Moreover, SCFAs can increase epithelial barrier functions, as measured by fluorescein isothiocyanate-dextran permeability assay, in a GPR43-dependent manner [25] or through the stabilization of hypoxia-inducible factor-alpha, particularly by butyrate [66]. Therefore, SCFAs can promote the barrier functions of the intestine, suggesting another protective role of butyrate against FA. In FA children compared to healthy subjects, different levels of fecal SCFAs, particularly butyrate, have been described [67,68,69]. As recently demonstrated, dysbiosis in *Faecalibacterium prausnitzii* is associated with AD, but it was shown that the presence of subspecies is more associated with AD than with the species overall [70]. Dysbiosis results in the suppression of high-butyrate-producer subspecies, leading to a reduction in overall butyrate production. Thus, different types of dysbiosis may share the same metabolic features leading to similar effects in term of SCFAs or of other metabolites levels that could facilitate the occurrence of FA. Interestingly, substantial correlations exist between the 16S rRNA profile, predicted metagenome, and metabolome of neonatal fecal samples, indicating a deterministic relationship between the bacterial community composition and metabolic microenvironment of the neonatal gut [29].It is also crucial that studies move beyond cataloguing of bacteria and toward functional characterization and mechanistic understanding. Metatranscriptomic studies will provide information regarding not only which bacteria and bacterial genes are present in a sample, but also the transcriptional activity of the community [71]. Metabolomics can reveal how bacterial metabolites facilitate interactions with the host and how they may influence the health state of the host [72,73]. Fine-level characterization of bacterial species can help reveal the function of the microbiome, which is affected by interactions among closely related bacteria that may compete for the same niche but have distinct activities. Together, these studies will provide a high-resolution picture of bacteria-host interactions that can lead to disease.Moreover, studies on germ-free mice may enable more precise determination of how microbial imbalances result in disease.

## 4. Modulation of the Gut Microbiota in FA

Primary prevention via microbiota-directed therapy is particularly appealing for potentially decreasing the incidence of FA. Children exposed to farm environments show a decreased risk for the development of allergic disease [74,75]. Although it has not been proven, a plausible explanation for the protective effect of early-life farm exposure is the role of microbiota, as individuals exposed to a farm environment exhibit a different microbial composition than those with other lifestyles [76]. Other epidemiologic factors protective against FA include having older siblings and pet exposure in early life [77]. Pet ownership is associated with a high microbial diversity in the home environment [78]. A recent study examining the influence of dietary patterns on the development of FA at the age of two years suggests that dietary habits influence FA development by changing the composition of the gut microbiota [43]. During infancy, breast milk provides various benefits to the new-born. Oligosaccharides, which are enriched in breast milk, favour the colonization of the SCFA-producing bacteria *Bifidobacterium* spp. [79]. Breast milk plays a critical role in the maturation of the gut microbiota by providing an initial source of commensal bacteria to the infant [80]. It has been recently demonstrated that higher levels of TGF-β2 in breast milk are associated with an increased relative abundance of several bacteria, including members of *Streptococcaceae* and *Ruminococcaceae*, and lower relative abundance of distinct *Staphylococcaceae* taxa [81]. One intervention that can modify the gut microbiota most significantly is diet, either by introducing new species or bacterial genes or by modulating the abundance of existing microbes in the community [82]. It has been demonstrated that an infant diet consisting of high levels of fruits, vegetables, and home-prepared foods was associated with fewer FA [43] (Figure 2). A high-fiber diet favours the outgrowth of bacteria capable of fermenting dietary fibers, such as *Bifidobacterium* and *Lactobacillus*, followed by an increase in serum SCFA levels. Neonatal prebiotic supplementation studies have failed to demonstrate any effect of prebiotics on the development of FA, but showed positive results for other allergic manifestations such as eczema [83].

Probiotics, defined as ingested microbes that provide health benefits to the host [84], may be beneficial by changing the microbiota. Recently published guidelines for atopic disease prevention from the World Allergy Organization concluded that there is a likely benefit to using probiotics in preventing eczema in children with a family history of allergic disease, but the evidence is very low in quality [85]. The most important factor in using probiotics against allergy is that this effect on the immune system is strain-specific. Thus, the results of studies for a selected bacterial strain cannot be adopted to other probiotic strains [84].Selected probiotics, such as *Lactobacillus rhamnosus* GG (LGG), were found to lower the risk of eczema when used by women during the last trimester of pregnancy, by breastfeeding mothers, or when given to infants [86].Studies examining the efficacy of currently available probiotics in treating FA have yielded conflicting results. It was recently demonstrated that oral immunotherapy supplemented with the probiotic *L. rhamnosus* CGMCC 1.3724 led to peanut unresponsiveness in 82% of allergic children [87]. In one randomized, double-blind, placebo-controlled study of infants with challenge-proven CMA, administration of *Lactobacillus casei* CRL431 and *Bifidobacterium lactis* Bb12 for 12 months did not affect the acquisition of tolerance to cow’s milk [88]. In contrast, we demonstrated in different studies that an extensively hydrolyzed casein formula (EHCF) containing LGG accelerated the development of tolerance acquisition in infants with CMA and reduced the incidence of other allergic manifestations [89,90,91]. When we compared the fecal microbiota of infants receiving this tolerance-inducing probiotic-supplemented formula to that obtained from infants receiving EHCF alone, we found significant positive correlations between the abundance of butyrate-producing genera, and an increase concentration of fecal butyrate [68]. The mechanisms of action of butyrate are multiple, but many of these involve epigenetic regulation of gene expression by inhibiting histone deacetylase (HDAC). Inhibition of HDAC 9 and 6 increased FoxP3 gene expression, and the production and suppressive function of Tregs [92]. We demonstrated that the use of EHCF+LGG induces stronger epigenetic regulation of Th1 and Th2 cytokines genes as revealed by the significantly different levels of promoter region methylation [93]. Similar results were obtained by examining the FoxP3 Treg-specific demethylated region (TSDR) methylation profile. FoxP3 TSDR demethylation and expression were significantly higher in children treated with EHCF+LGG compared to in children treated with other dietary strategies [94]. These results strongly suggest that acting on gut microbiota composition and function can have long-term protective effects in children with FA.

## 5. Conclusions

Our understanding of the role of gut microbiota in the development of FA continues to evolve. Larger studies, preferably longitudinal birth cohort studies with more homogenous designs, are needed to clarify the presence or absence of a defined dysbiotic signature associated with FA. In addition, larger interventional trials are required to evaluate the roles of different probiotic strains in modulating gut microbiota composition and function. Integrating these findings with epigenetics and metabolomics data enable the development targeted microbiota with innovative approaches to prevent and manage FA.
